# Laparoscopic Management of Gastric Band Migration with Acute Gastric Perforation — a Video Vignette

**DOI:** 10.1007/s11695-022-06194-7

**Published:** 2022-07-09

**Authors:** Sebastian Holländer, Gereon Gäbelein, Antonios Spiliotis, Philipp Robert Scherber, Matthias Glanemann

**Affiliations:** 1 Department of General Surgery, Vascular-, Visceral- and Pediatric Surgery, Saarland University Medical Center, Kirrberger Str, 66421 Homburg, Germany; 2 Department of Surgery, Charité Universitätsmedizin Berlin, Charitépl. 1, 10117 Berlin, Germany

**Keywords:** Gastric banding, Gastric band erosion, Gastric band perforation, Complications after gastric banding

## Introduction

Laparoscopic adjustable gastric banding (LAGB) was once considered to be a safe and effective surgical treatment for morbid obesity. Over the past years, its long-term efficacy and safety came into question by the occurrence of complications such as intragastric band migration. The incidence of intragastric band migration is reported to be between 0.4 and 3.8% [1–4]. The incidence of gastric perforations is 0.1–0.8% [5–7]. Removal of the band in case of migration is always required and often possible via upper endoscopy [2]. Overall, septic complications are rare. However, when they do occur they can be life-threatening.

## Purpose

The purpose of this video was to demonstrate the feasibility of a laparoscopic approach in case of acute gastric band perforation in a 52-year-old male patient who underwent LAGB for morbid obesity 20 years ago (initial weight 146 kg; BMI 45.56 kg/m^2^; current weight 86 kg; BMI 26.84 kg/m^2^). The patient was transferred to our institution due to an acute abdomen, fever, and dysphagia.

## Methods

We provide an intraoperative video to demonstrate the feasibility of a laparoscopic approach in a septic constellation. The video shows the laparoscopic removal of the LAGB and the closure of the gastric defect by partial fundus resection.

## Results

The procedure started with the blunt preparation of omental fat covering the left subphrenic area. The lesser sac was opened by dissecting the short gastric vessels towards the gastric fundus, revealing the band lock and the perforation site. After removal of the gastric band, the gastric fundus was completely mobilized, and the left crus of the diaphragm was identified. By use of two stay sutures at the corners of the gastric defect, it was possible to completely resect the perforated area with two 60-mm endo-stapler cartridges. Interrupted resorbable sutures were placed over the staple line. Intraoperative upper endoscopy showed no remaining defect, and no air leakage was detected.

The operative time was 150 min. The postoperative course was uneventful. Upper GI contrast study on POD 3 showed no signs of leakage. The patient was discharged on postoperative day 7. At short-term follow-up, the patient presented asymptomatic 3, 6, and 12 months after the procedure.

## Discussion

Major complications of LAGB such as gastric perforations are rare. While removal of migrated gastric bands is often possible via upper endoscopy, surgical treatment in case of acute perforation and subsequent peritonitis is mandatory.

In the presented case, the perforation was located at the gastric fundus distant from the angle of His. In case of a “favorably” located perforation — as in the presented video — resection of the perforation area using an endo-stapler can be considered. For more inconveniently located perforations, surgeons should also have additional options in their armamentarium such as suturing, omental patch, fundoplication, Foley catheterization [8] or resection and gastro-gastrostomy [9].

## Conclusion

Acute gastric perforation following gastric banding is a rare but severe complication. In such cases, depending on the localization of the perforation site, a minimally invasive partial gastric resection should be considered, even in a septic constellation.

## Supplementary Information

Below is the link to the electronic supplementary material.

Video1 (MP4 430 MB)
